# PRDM16 expression and function in mammalian cochlear development

**DOI:** 10.1002/dvdy.480

**Published:** 2022-05-07

**Authors:** Michael Ebeid, Kathy Barnas, Hongji Zhang, Amal Yaghmour, Gabriele Noreikaite, Bryan C. Bjork

**Affiliations:** ^1^ College of Graduate Studies, Midwestern University Downers Grove Illinois USA; ^2^ Department of Anatomy Midwestern University Downers Grove Illinois USA; ^3^ Chicago College of Osteopathic Medicine Midwestern University Downers Grove Illinois USA; ^4^ Biomedical Sciences Program Midwestern University Downers Grove Illinois USA

**Keywords:** Cochlear development, Kölliker's organ, PRDM16

## Abstract

**Background:**

PR domain containing 16 (PRDM16) is a key transcriptional regulator in the development of craniofacial, adipose, and neural tissues. Our lab identified PRDM16 expression in the epithelial cells of the Kölliker's organ (KO) that starts at ~E13.5 and is maintained until KO disappearance. A transgenic mouse model that carries a gene trap null allele of *Prdm16* (*Prdm16* ^*cGT*^) was used to characterize the impact of *Prdm16* loss on cochlear development.

**Results:**

At P0 *Prdm16* ^*cGT*^ null cochlea exhibited hypoplastic KO, shortened cochlear duct, increased density of hair cells (HCs) and supporting cells (SCs) in the apical turn as well as multiple isolated ectopic HCs within the KO domain. KO epithelial cells proliferation rate was reduced in the apical turn of the developing *Prdm16* ^*cGT*^ null cochlea vs controls. Bulk RNA sequencing of cochlear duct cells at E14.5 followed by quantitative real time PCR and mRNA Fluorescence in‐situ hybridization (FISH) validation identified differentially expressed genes in *Prdm16* ^*cGT*^ null vs littermate control cochleae. Upregulated genes at E14.5 included *Fgf20*, as well as several Notch pathway genes (*Lfng*, *Hes1*, and *Jag1*).

**Conclusions:**

This study characterizes *Prdm16* expression during cochlear development and establishes its requirement for KO development.

## Key Findings

- Prdm16 is a novel marker for Kölliker's organ in the developing mammalian cochlea
- Prdm16 is required for Kölliker's organ cell proliferation and is involved in spiral limbus and tectorial membrane development
- Prdm16 inhibit sensory fates in Kölliker's organ cells during development

## 1 INTRODUCTION

Mammalian cochlear development involves a precisely orchestrated series of events that convert a simple thickened epithelium (otic placode) to a complex structure connected to the central nervous system. In mice, the cochlear duct arises from the otocyst around embryonic day 11 (E11).^1^ As the cochlear duct extends and coils, a subset of cells within its floor begins to develop as the prosensory domain that will give rise to the organ of Corti.^2^ This domain is localized to a narrow strip extending along the cochlear duct floor.^1^ On the neural side of the developing organ of Corti, a group of epithelial cells constitutes Kölliker's organ (KO). This organ is a transient epithelial structure that undergoes remodeling during the embryonic and early postnatal stages. As the cochlea matures, KO columnar cells are replaced by cuboidal cells lining the mature inner sulcus and represent approximately 12% of the original cell count.^3^ Thyroid hormone has been shown to regulate this process as its deficiency leads to prolonged survival of KO cells and malformed tectorial membrane.^4^, ^5^ The role of KO is still under investigation and is thought to be involved in tectorial membrane formation^6^ and generating intrinsic spontaneous activity that drives primary afferent auditory neurons.^7^, ^8^ Previous studies have shown that KO epithelial cells have some capacity to generate sensory hair cells upon forced expression of genes involved in hair cell differentiation such as *Atoh1*.^9^, ^10^, ^11^ The mechanism underlying the regulation of KO cells' capacity to generate hair cells is still unclear. It has been proposed that the Notch signaling pathway restricts the sensory fate at the neural boundary of the developing organ of Corti,^12^ while Hedgehog signaling represses the sensory competence of the KO cells.^13^ Molecular mechanisms governing KO development, sensory competence, and the ultimate function of KO in the mature cochlea are still under investigation.

Using single‐cell transcriptomic analysis and immunostaining, we identified that PR domain‐containing 16 (*Prdm16*) is expressed within KO during cochlear development. This gene encodes a protein that belongs to the PRDM family which consists of 17 members and is characterized by the combination of a PR‐SET domain and a number of Zn‐finger domains.^14^ The PR domain exhibits Histone‐H3 monomethylase activity, while zinc‐finger domains are capable of sequence‐specific DNA binding.^15^ *Prdm16* has been identified as a key regulator in the development of multiple diverse cell types, including neuronal stem cells,^16^ hematopoietic stem cells,^17^ craniofacial,^18^, ^19^ and adipose tissues.^20^ A few molecular mechanisms were identified downstream of *Prdm16*, including repression of transforming growth factor beta (TGF‐β) superfamily signaling during craniofacial development^18^, ^21^ and chromatin remodeling activity during brown adipose tissue development.^22^ The role of *Prdm16* in mammalian cochlea development, and ultimately in hearing function, has not been investigated. *PRDM16* is located at 1p36.32 in the human genome (hg38), and deletion of genetic material from the short (p) arm of chromosome 1 lead to 1p36 deletion syndrome.^23^ Sensorineural hearing impairment was reported in 82% of patients with 1p36 deletion syndrome,^24^ yet the underlying gene responsible for hearing impairment in 1p36 deletion syndrome has not been identified. This study describes the expression of *Prdm16* during mammalian cochlear development and the developmental consequences of its loss.

## 2 RESULTS

**FIGURE 1:**
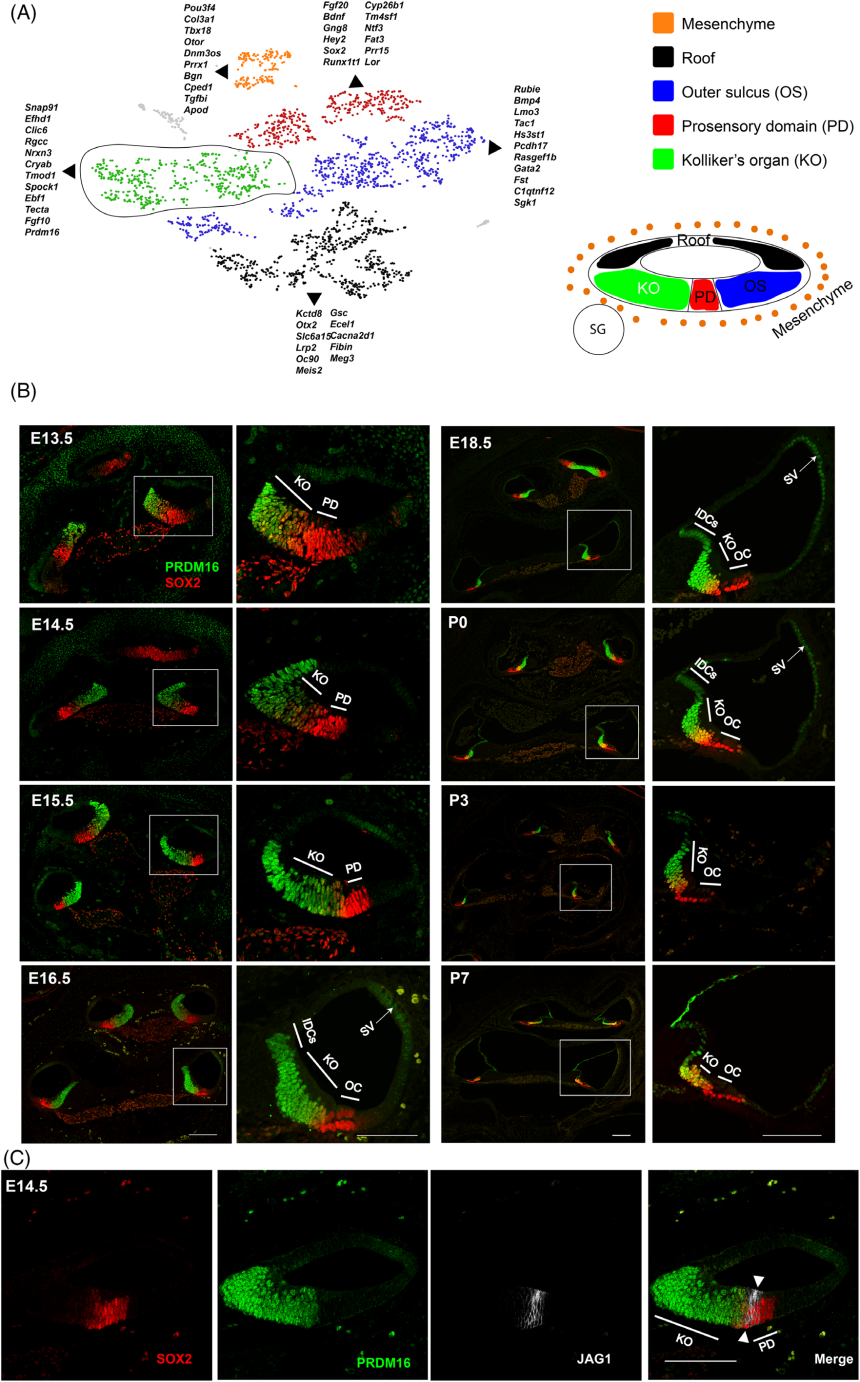
*Prdm16* expression marks the KO during mouse cochlear development. (A) t‐SNE plot representing graph‐based clustering of single cell gene expression in WT E14.5 isolated cochlear duct cells showing different cell clusters (color‐coded) and enriched gene sets per cluster. (B) Immunostaining of WT cochlear ducts for PRDM16 (green) and SOX2 (red) at multiple time points (E13.5‐P7) showing nuclear expression of *Prdm16* within KO epithelial cells. White boxes indicate magnified middle turn at each time point. (C) Immunostaining of E14.5 WT cochlear section (middle turn) showing a clear demarcation between JAG1 and PRDM16 expression (white arrowheads). IDCs, interdental cells; KO, Kölliker's organ; OC, organ of Corti; PD, prosensory domain; SG, spiral ganglion; SV, stria vascularis (scale bar = 100 μm).

To identify novel genes required for mouse cochlear development, we utilized the 10× genomics platform and bioinformatics analysis to capture and analyze individual single‐cell transcriptome profiles from WT cochlear duct cells at E14.5. Graph‐based clustering was used to identify clusters of cells with similar transcriptional profiles. We utilized previously identified markers for different populations of cells including mesenchyme markers *Tbx18* & *Pou3f4*,^25^, ^26^ non‐sensory roof epithelial markers *Fgf9*, *Otx1* & *Otx2*,^27^, ^28^ prosensory domain markers *Sox2*, *Jag1* & *Hey2*,^29^, ^30^, ^31^ KO markers *Fgf10* & *tecta* ^32^ and future outer sulcus markers *Bmp4* & *Lmx1a* ^1^ to cluster different population of cells within the developing cochlear duct (Figure 1A). To uncover novel genes expressed within each population, we performed differential gene expression analysis across known populations. A list of top 20 differentially expressed genes within each population is shown in Table 1 and the whole data set is shown in Table S1. Analysis of differentially expressed genes within the KO population at E14.5 identified *Prdm16* as a novel marker for this population (Figure 1A). Next, we characterized the expression of *Prdm16* in WT cochleae through immunostaining using anti‐PRDM16 antibody^33^ concomitantly with anti‐SOX2 staining as established marker for the prosensory domain and supporting cells later in development. Our data confirmed PRDM16 expression within the nuclei of KO cells as early as E13.5, and its expression is maintained in the KO domain throughout development until postnatal day 7 (P7) (Figure 1B). Expression appeared first in the basal and middle regions of E13.5 cochlear duct then extended to the cochlea's apical turn around E15.5. We observed partial overlap between the PRDM16 and SOX2 domain at all time points in the region of inner supporting cells. PRDM16 staining shows moderate level of expression within the interdental cells (IDCs) and low level of expression within the stria vascularis (SV) (Figure 1B). Such expression is maintained until P0 when it rapidly declines. The KO start diminishing in size at P0 while PRDM16 expression is maintained, and the whole organ disappears by ~P10, leaving only inner sulcus cells. Since JAG1 is known to be restricted to the prosensory domain around E14.5, we performed co‐staining with PRDM16 and confirmed that PRDM16 expression is limited to the KO, since there is no co‐localization of PRDM16 and JAG1 (Figure 1C).

**TABLE 1:** Top 20 enriched genes within each cluster at E14.5 in WT mouse cochlea.

Kölliker's organ Genes	L2FC	*P* value	Prosensory domain Genes	L2FC	*P* value	Outer sulcus Genes	L2FC	*P* value	Roof Genes	L2FC	*P* value	Mesen‐chyme Genes	L2FC	*P* value
*150 001‐5O10Rik*	4.50	2.18E‐50	*Fgf20*	4.31	1.35E‐64	*Rubie*	4.62	1.46E‐67	*Kctd8*	6.35	5E‐124	*AI593442*	9.83	2.07E‐42
*Snap91*	4.20	8.49E‐61	*Bdnf*	3.82	1.36E‐45	*Bmp4*	4.32	4.70E‐83	*Otx2*	6.14	2.4E‐137	*Pou3f4*	8.99	8.64E‐96
*Efhd1*	3.64	1.58E‐51	*Gng8*	3.47	1.38E‐39	*Lmo3*	4.18	7.90E‐63	*Slc6a15*	5.91	3.98E‐95	*Col3a1*	8.99	1.47E‐92
*Clic6*	3.26	3.86E‐40	*Hey2*	3.42	2.65E‐47	*Tac1*	3.40	1.67E‐37	*Lrp2*	5.91	2.69E‐70	*Tbx18*	8.67	7.12E‐123
*Rgcc*	2.70	6.94E‐30	*Sox2*	3.37	3.08E‐49	*Hs3st1*	2.68	2.43E‐35	*Oc90*	5.58	7.2E‐142	*Otor*	8.50	1.71E‐97
*Nrxn3*	2.60	1.78E‐23	*Runx1t1*	3.27	8.18E‐32	*Pcdh17*	2.47	6.37E‐23	*Meis2*	5.53	8.3E‐99	*Dnm3os*	8.32	1.88E‐67
*Cryab*	2.50	3.07E‐23	*Cyp26b1*	3.13	1.41E‐34	*Rasgef1b*	2.38	1.66E‐26	*Gsc*	5.49	5.85E‐93	*Prrx1*	8.00	2.23E‐78
*Tmod1*	2.49	7.62E‐26	*Tm4sf1*	2.94	8.14E‐31	*Gata2*	2.36	4.27E‐29	*Ecel1*	5.20	1.12E‐83	*Bgn*	7.55	1.63E‐69
*Spock1*	2.48	6.5E‐22	*Ntf3*	2.80	4.26E‐34	*Fst*	2.29	1.80E‐25	*Cacna2d1*	4.95	9.64E‐84	*Cped1*	7.23	5.27E‐72
*Ebf1*	2.47	6.32E‐27	*Fat3*	2.73	1.18E‐29	*C1qtnf12*	2.10	7.49E‐23	*Fibin*	4.85	1.8E‐82	*Tgfbi*	7.08	7.47E‐60
*Tecta*	2.46	4.3E‐26	*Prr15*	2.60	8.08E‐23	*Sgk1*	2.01	6.88E‐19	*Meg3*	4.35	1.35E‐89	*Apod*	7.07	1.71E‐35
*Fgf10*	2.35	6.02E‐22	*Lor*	2.58	2.25E‐24	*Itih5*	1.99	5.81E‐20	*Fut9*	4.34	2.53E‐73	*Pdgfra*	6.76	5.80E‐57
*Prdm16*	2.30	8.07E‐19	*Cdkn1b*	2.54	3.01E‐26	*Rprm*	1.80	3.20E‐16	*Tbx1*	4.30	2.43E‐78	*Col26a1*	6.08	6.31E‐65
*Dcn*	1.97	1.33E‐13	*Tectb*	2.47	6.48E‐24	*Gata3*	1.77	1.57E‐17	*Mme*	4.19	1.21E‐72	*Col1a2*	6.05	4.84E‐57
*Chst15*	1.95	2.7E‐15	*S100a1*	2.46	1.13E‐24	*Unc5b*	1.76	1.23E‐15	*Krt19*	4.07	9.85E‐65	*Eva1b*	5.92	2.52E‐60
*Cenpf*	1.86	1.79E‐11	*Lgr5*	2.40	4.56E‐23	*Smad7*	1.73	6.66E‐16	*Ap1s2*	3.91	2.26E‐62	*Clmp*	5.39	7.04E‐45
*Top2a*	1.78	1.03E‐11	*Sox21*	2.40	5.58E‐24	*Ppfibp1*	1.73	3.10E‐15	*Pid1*	3.78	8.18E‐64	*Car3*	4.94	1.62E‐24
*Mia*	1.78	1.58E‐13	*Rassf9*	2.38	1.14E‐23	*Sfrp1*	1.68	1.00E‐15	*Ehbp1*	3.60	6.29E‐56	*Atp1a2*	4.69	1.14E‐33
*Tectb*	1.76	1.91E‐11	*Ntm*	2.25	3.59E‐20	*923 010‐2O04Rik*	1.65	2.16E‐14	*Lurap1l*	3.54	6.64E‐59	*Foxc1*	4.51	6.65E‐35
*Cdh4*	1.74	1.46E‐11	*Hs6st2*	2.24	4.43E‐20	*Angpt1*	1.58	4.14E‐12	*Phldb2*	3.36	4.07E‐49	*Dab2*	4.37	2.74E‐22

**FIGURE 2:**
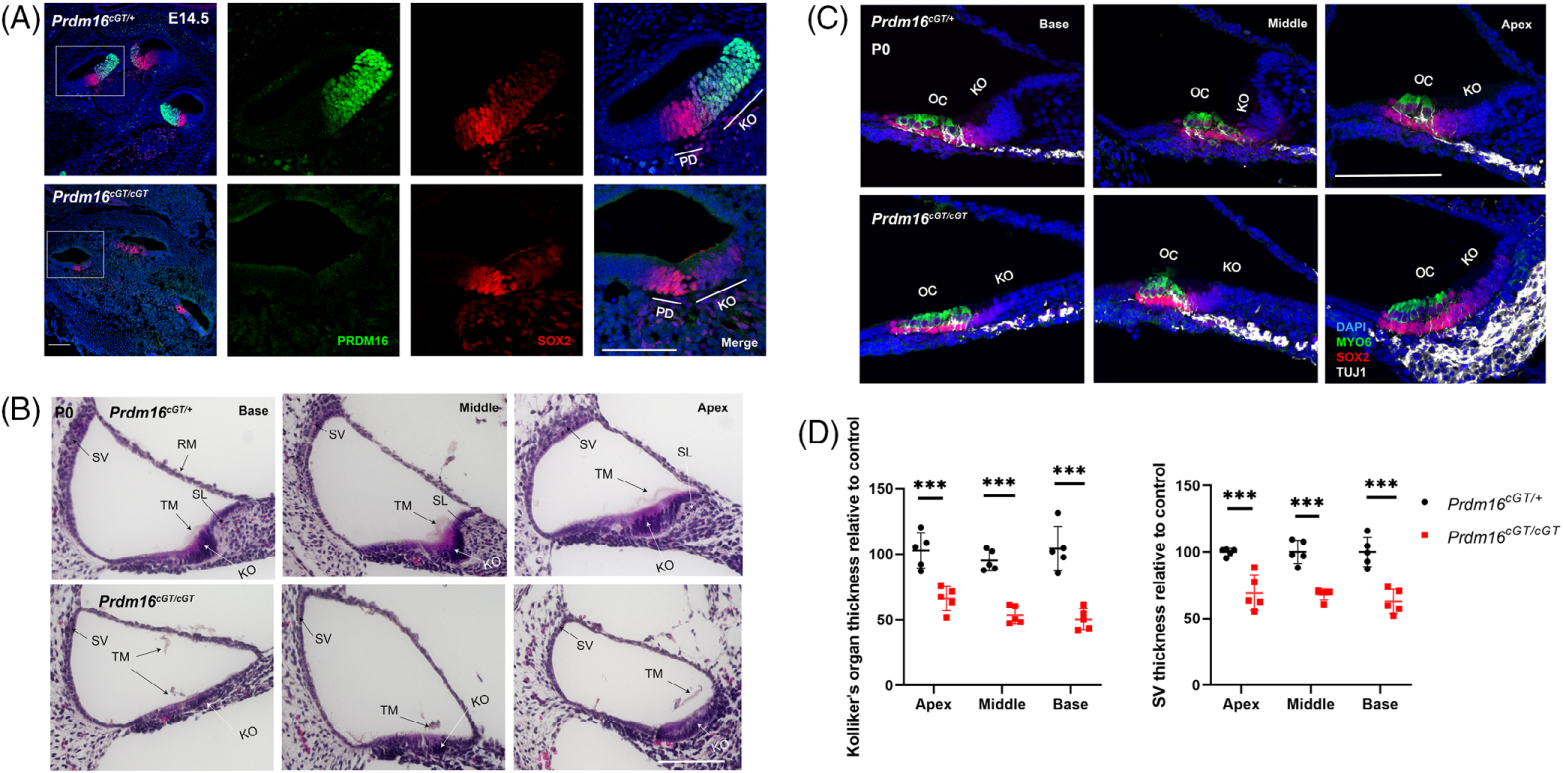
*Prdm16* is required for normal development of multiple cochlear structures including KO, spiral limbus, stria vascularis and tectorial membrane. (A) Validation of *Prdm16* deletion in cochlear duct of *Prdm16* ^*cGT*^ mouse model by immunostaining for PRDM16 (green) and SOX2 (red) at E14.5 showing lack of PRDM16 signal in *Prdm16* ^*cGT*^ null cochlear sections. White boxes indicate magnified turns. (B) H&E staining of cochlear duct sections from *Prdm16* ^*cGT*^ null (*Prdm16* ^*cGT/cGT*^) and littermate heterozygote controls (*Prdm16* ^*cGT/+*^) pups at P0 showing hypoplastic KO & spiral limbus with detached tectorial membrane and thin stria vascularis. (C) Immunostaining of cochlear duct sections from *Prdm16* ^*cGT*^ null and littermate heterozygote controls at P0 showing DAPI, MYO6 (hair cell marker), SOX2 (supporting cell marker), TUJ1 (neuronal marker) staining showing high HC density in the apical turn of *Prdm16* ^*cGT*^ null cochlea. (D) Graphs showing average KO thickness and SV thickness at P0 in *Prdm16* ^*cGT*^ null relative to heterozygous controls at each turn (n = 5 per group, Mean ± SD, multiple Student's *t*‐tests, *P*‐value *** < 0.001). KO, Kölliker's organ; OC, organ of Corti; PD, prosensory domain; RM, Reisner's membrane; SV, stria vascularis; SL, spiral limbus; TM, tectorial membrane (scale bar = 100 μm).

To understand the role of *Prdm16* in cochlear development, we used the *Prdm16* ^*cGT*^ gene trap null mutant mouse strain^33^ and validated the loss of PRDM16 in the cochlea by immunohistochemistry (Figure 2A). Since *Prdm16* ^*cGT*^ null mutants die shortly after birth due in part to complications related to presence of cleft secondary palate, we characterized the cochlear phenotype at P0. H&E and immunostaining of cochlear sections at P0 showed hypoplastic KO in *Prdm16* ^*cGT*^ null cochlea compared with heterozygote littermate controls (*Prdm16* ^*cGT/+*^) (Figure 2B,C). The thickness of KO was significantly reduced across the whole length of the cochlear duct (Student's *t*‐test *P* value <0.001, n = 5) (Figure 2B‐D). We also observed failed development of the spiral limbus (SL), along with detachment and fragmentation of tectorial membrane (TM) across all cochlear turns in *Prdm16* ^*cGT*^ null mice (Figure 2B‐D). Additionally, SV thickness was significantly reduced across all cochlear turns in *Prdm16* ^*cGT*^ null cochlea (Student's *t*‐test *P*‐value <0.001, n = 5) (Figure 2D). We did not observe any difference between *Prdm16* heterozygotes and WT cochlea.

**FIGURE 3:**
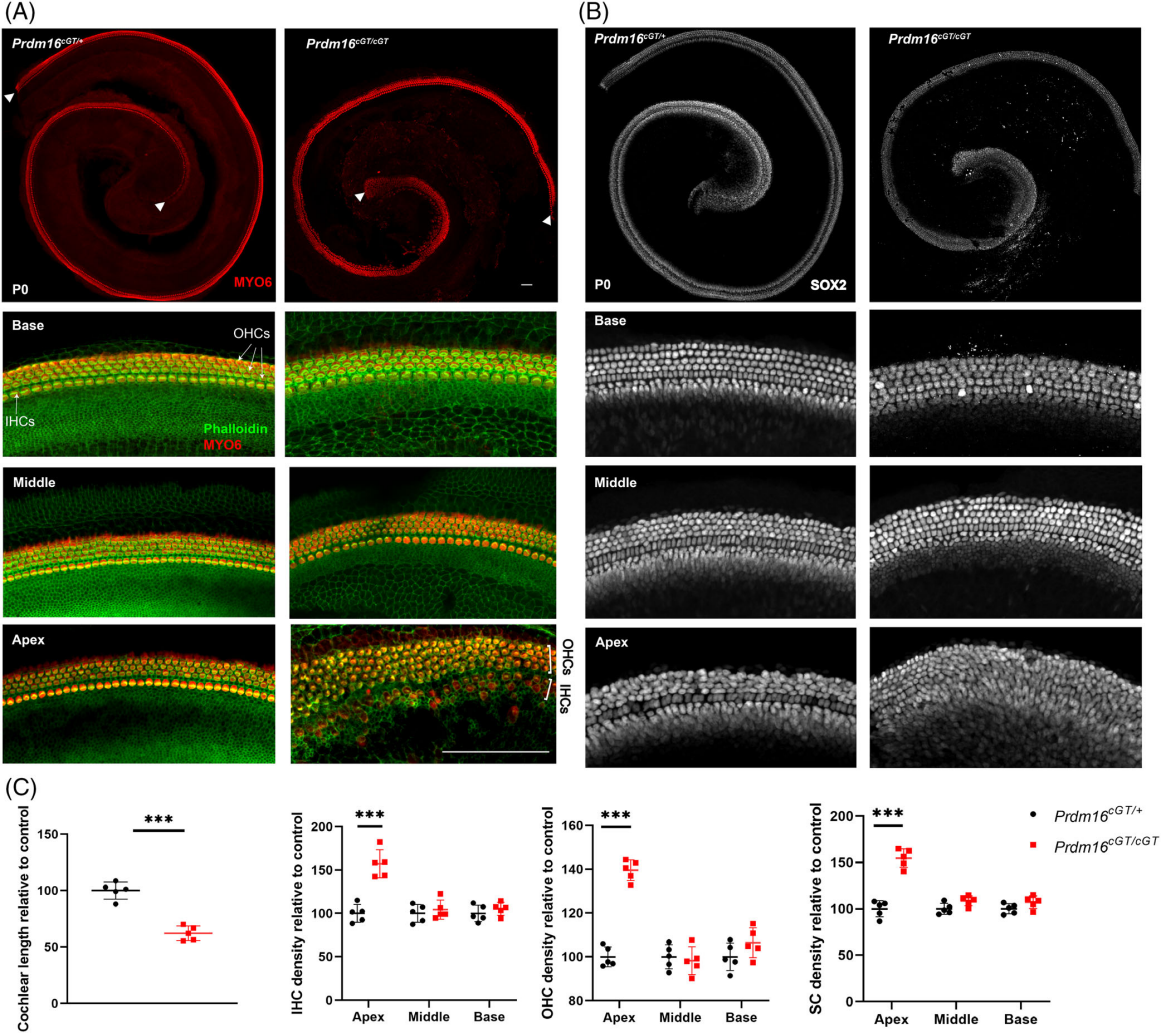
*Prdm16* ^*cGT*^ Null mice exhibit short cochlear duct and high density HCs & SCs in the apical turn at P0. Whole‐mount immunostaining of cochlear epithelium from P0 *Prdm16* ^*cGT/cGT*^ and littermate heterozygote controls stained with hair cell marker (MYO6) and F‐actin enriched stereocilia bundles marker (phalloidin) in (A) and supporting cell marker (SOX2) in (B). Arrowheads show the beginning and the end of cochlear length measurement. Scale bar = 200 μm. (C) Quantification of cochlear length, IHC, OHC, and SC density in different cochlear turns at P0 (n = 5 per group, Mean ± SD, multiple Student's *t* tests, *P* value *** < 0.001). IHC, Inner hair cells; OHC, outer hair cells, SC: Supporting cells.

**FIGURE 4:**
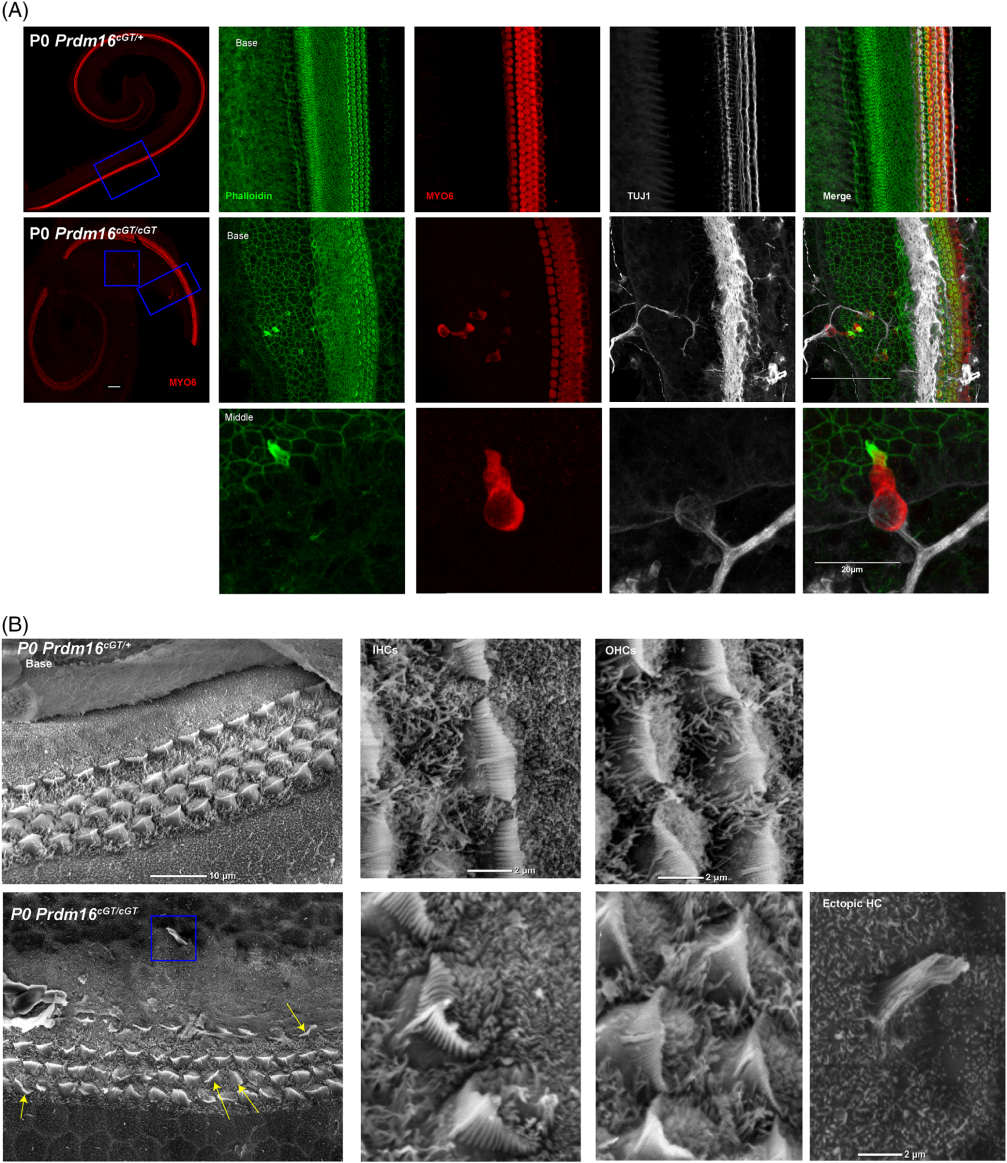
*Prdm16* ^*cGT*^ null cochlea shows ectopic HCs within the KO. (A) Whole‐mount immunostaining of cochlear epithelium from P0 *Prdm16* ^*cGT/cGT*^ and littermate heterozygote controls stained with hair cell marker (MYO6), F‐actin enriched stereocilia bundles marker (phalloidin), neuron marker (TUJ1) showing no ectopic HCs outside the organ of Corti in controls (top panel), while *Prdm16* ^*cGT*^ null cochlea shows either cluster of ectopic HCs or individual ectopic HC in the KO region. Ectopic HCs are innervated as shown with TUJ1 staining. (B) Scanning electron microscopy of cochlear epithelium from P0 *Prdm16* ^*cGT/cGT*^ and littermate heterozygote controls showing stereocilia bundles of IHCs, OHCs and ectopic HC. Within the *Prdm16* ^*cGT*^ null cochlea, some native IHCs and OHCs showed rotated axis of stereocilia bundles (yellow arrows). Blue boxes mark the magnified regions. Scale bar = 200 μm unless otherwise specified.

To test if the organ of Corti (OC) is impacted by *Prdm16* loss we performed whole mount immunostaining with markers of HCs (MYO6) and SCs (SOX2). *Prdm16* ^*cGT*^ null mutants exhibit shortened cochlear ducts (62% compared with heterozygote control cochleae) (Student's *t*‐test *P* value <0.001, n = 5) (Figure 3A‐C), and the density of HCs and SCs per 100 μm of cochlear length was significantly increased in the apical turn (IHC and SC density around 150% relative to controls, and OHC around 140% relative to controls) (Student's *t*‐test *P* value <0.001, n = 5) (Figure 3B‐C). Taken together, shortening of the cochlear duct and increased density of HCs and SCs in the apical turn point to a defect in the extension of the apical cochlear turn in *Prdm16* ^*cGT*^ null cochlea. Interestingly, we also identified ectopic HCs within the KO in *Prdm16* ^*cGT*^ null cochlea that are MYO6‐positive and exhibit stereocilia bundles, as evident by phalloidin staining (Figure 4A). The number of ectopic HCs in the whole cochlea ranged from 6 to 32 cells/cochlea (N = 5) and they occurred either in clusters of 4 to 5 cells or as individual cells within the KO (Figure 4A). Immunostaining for the neuronal marker, TUJ1, revealed that 100% of the ectopic HCs are innervated with nerve fibers running through the KO (Figure 4A). The distance between ectopic HCs and native IHCs ranged from 38 to 98 μm. Ectopic HCs appeared to have pear‐shaped cell body, with basal nucleus, apical stereocilia bundle and a calyx afferent surrounding the basolateral membrane as evident by TUJ1 staining (Figure 4A). Scanning electron microscopy of the stereocilia bundles of ectopic HCs shows cylindrical arrangement of multiple rows of stereocilia compared with the U or V‐like arrangement of stereocilia in native HCs at P0 (Figure 4B). Within the *Prdm16* ^*cGT*^ null cochlea, some native IHCs and OHCs showed stereocilia bundles with rotated axes at P0 (Figure 4B).

**FIGURE 5:**
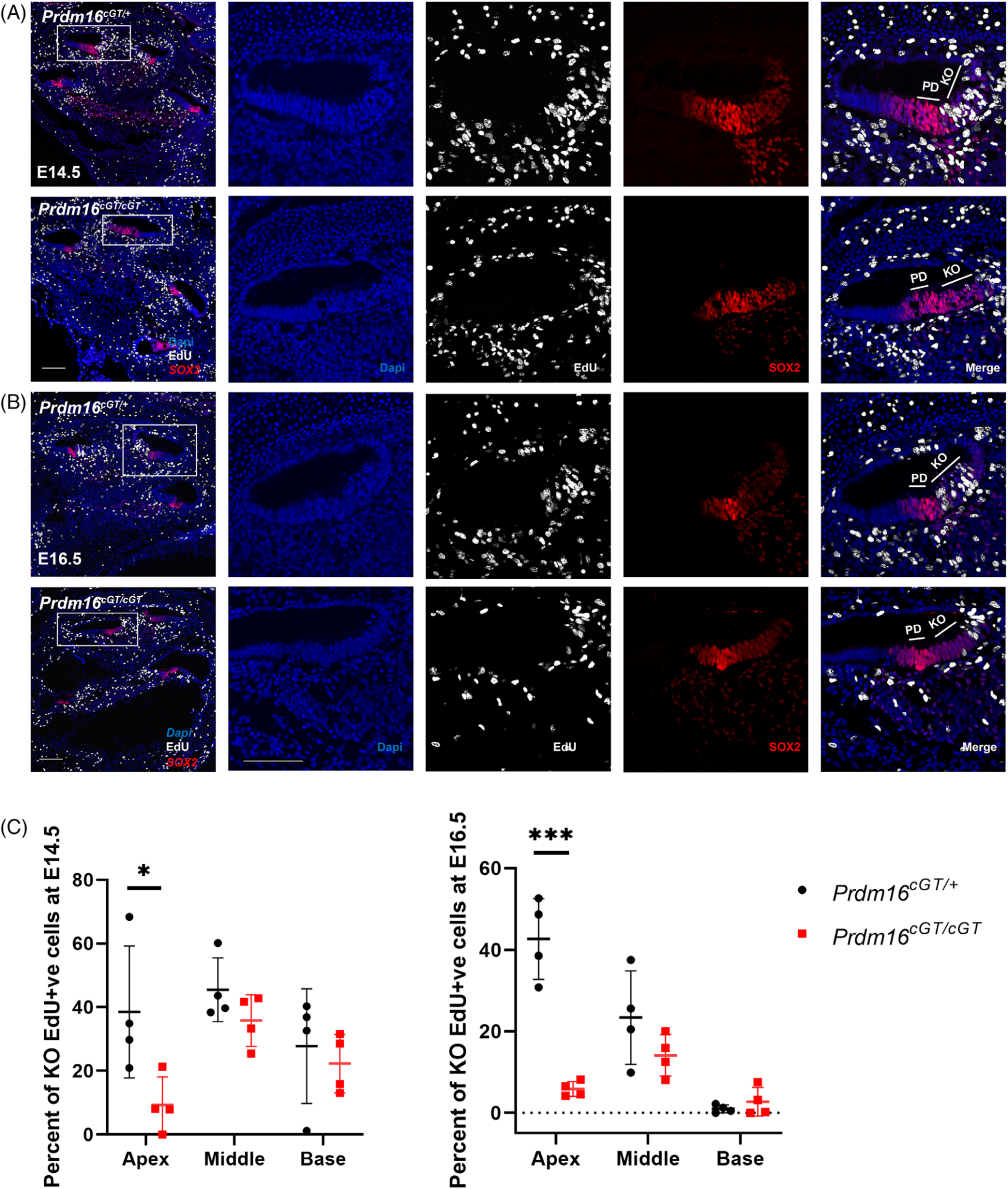
*Prdm16* regulates KO cell proliferation during development. Immunostaining of cochlear sections from *Prdm16* ^*cGT/cGT*^ and littermate heterozygote controls showing EdU (proliferation marker), DAPI and SOX2 (prosensory domain marker) staining in the apical turn at E14.5 (A) and E16.5 (B). Scale bar = 100 μm. White boxes mark the magnified regions (KO, Kölliker's organ; PD, prosensory domain). (C) Percentage of KO cells incorporating EdU at E14.5 and E16.5 for *Prdm16* ^*cGT/cGT*^ and littermate heterozygous controls at each turn (*n* = 4 per group, Mean ± SD, multiple Student's *t* tests, *P* value * < 0.05, *** < 0.001).

To understand the possible mechanism underlying the hypoplastic KO and short cochlear duct in *Prdm16* ^*cGT*^ null cochlea, we assessed the proliferation of KO cells during cochlear development at E14.5 and E16.5 in *Prdm16* ^*cGT*^ null mutant compared with heterozygote littermates using EdU proliferation marker. The percentage of KO cell incorporating EdU proliferation marker was significantly reduced in apical turns of *Prdm16* ^*cGT*^ null cochlea vs controls at both E14.5 (Student's *t*‐test *P* < 0.05, n = 4) and E16.5 (Student's *t*‐test *P* < 0.001, n = 4) (Figure 5A‐C). We noticed that by E16.5, KO cells in the base have already stopped proliferating.

**FIGURE 6:**
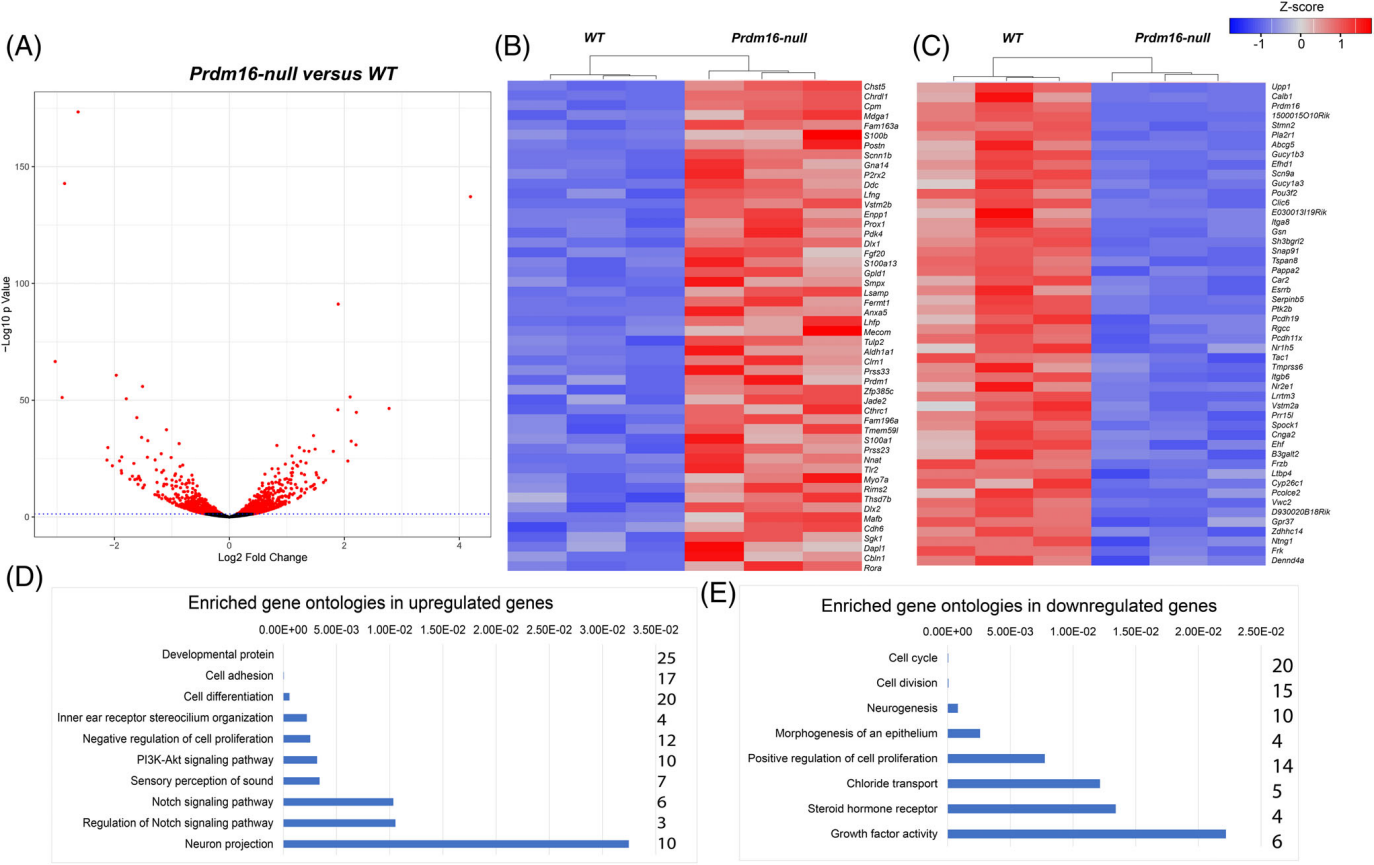
RNA sequencing detects differentially expressed genes (DEGs) in *Prdm16* ^*cGT*^ null cochlear duct at P0. (A) Volcano plot showing DEGs with statistical significance in red. (B) Heatmap of top 50 DEGs that show upregulation in *Prdm16* ^*cGT*^ null vs WT. (C) Heatmap of top 50 DEGs that show downregulation in *Prdm16* ^*cGT*^ null vs WT (D, E) Graphs representing enriched gene ontologies in DEGs (*P* values are shown on the x‐axis on top). The number of genes within each category is shown on the right side of each graph. (n = 3 per group, Waldic Statistics, FDR *P* value <0.05).

To investigate the molecular mechanisms underlying the role of *Prdm16* during cochlear development, we collected bulk RNA from three *Prdm16* ^*cGT*^ null, three heterozygote, and three WT E14.5 cochlear ducts. Analysis of RNA sequencing revealed downregulation of 277 genes and upregulation of 218 genes in *Prdm16* ^*cGT*^ null cochleae compared with WT (Log2 fold change >0.5, FDR adjusted *P* value <0.05, n = 3) (Figure 6A‐C). The top 50 differentially expressed genes are shown in Table 2, and the whole data set is shown in Table S2. We cross‐referenced differentially expressed genes to domain‐specific genes from our scRNA sequencing data at E14.5 (Figure 1A and Table S1). Interestingly, upregulated genes included multiple prosensory‐specific genes such as *Fgf20*, *S100a1*, *Lor*, *Lfng*, *Jag1*, and *p27Kip1* (*Cdkn1b)*. We also noticed upregulation of two Prdm family genes in response to *Prdm16* loss; *Mecom* (*Prdm3*) and *Prdm1* indicating a compensatory response which has been shown previously.^19^ Among the downregulated genes were KO‐specific genes, including *Efhd1*, *Itga8*, *Gsn*, *Clic6*, *Fgf10*, and *Tecta*. We utilized the Database of Annotation, Visualization and Integrated Discovery (DAVID)^34^, ^35^ to uncover enriched gene ontologies within upregulated and downregulated genes. We found multiple Notch signaling pathway genes enriched within the upregulated gene set including *Lfng*, *Postn*, *Jag1*, and *Hes1* (Figure 6D). Enriched gene ontologies within downregulated genes included positive regulation of proliferation and chloride transport (Figure 6E).

**TABLE 2:** Top 50 up‐and downregulated genes in *Prdm16* ^*cGT*^ null cochlear duct compared to WT control at E14.5 (RNA sequencing data).

Downregulated gene symbol	Log2 fold change	FDR adj *P* value	Upregulated gene symbol	Log2 fold change	FDR adj *P* value
*Upp1*	−3.03095	8.65E‐64	*Chst5*	2.208205	1.88E‐42
*Calb1*	−2.91107	1.21E‐48	*Chrdl1*	1.892087	2.88E‐88
*Prdm16*	−2.86781	1.24E‐139	*Cpm*	1.887978	1.72E‐43
*1500015O10Rik*	−2.63236	5.64E‐170	*Mdga1*	1.550947	3.40E‐15
*Stmn2*	−2.1146	1.22E‐27	*Fam163a*	1.49125	5.46E‐27
*Pla2r1*	−1.96931	5.29E‐58	*S100b*	1.475811	1.60E‐12
*Abcg5*	−1.91204	4.17E‐22	*Postn*	1.462296	1.41E‐32
*Gucy1b3*	−1.87374	9.84E‐24	*Scnn1b*	1.384264	4.12E‐26
*Efhd1*	−1.79445	3.69E‐48	*Gna14*	1.332706	2.95E‐21
*Scn9a*	−1.73202	1.31E‐14	*P2rx2*	1.305185	2.03E‐16
*Gucy1a3*	−1.68744	7.69E‐16	*Ddc*	1.300187	3.01E‐26
*Pou3f2*	−1.66321	4.48E‐21	*Lfng*	1.252448	5.57E‐17
*Clic6*	−1.61144	3.08E‐40	*Vstm2b*	1.239903	6.31E‐22
*E030013I19Rik*	−1.60824	1.03E‐14	*Enpp1*	1.217822	1.22E‐27
*Itga8*	−1.5675	4.40E‐15	*Prox1*	1.187037	5.91E‐20
*Gsn*	−1.54794	2.85E‐14	*Pdk4*	1.174113	3.36E‐11
*Sh3bgrl2*	−1.52764	7.75E‐32	*Dlx1*	1.171678	2.46E‐20
*Snap91*	−1.51172	2.81E‐53	*Fgf20*	1.148081	1.63E‐12
*Tspan8*	−1.42692	1.32E‐23	*S100a13*	1.122683	6.38E‐09
*Pappa2*	−1.41921	1.87E‐30	*Gpld1*	1.113356	2.17E‐21
*Car2*	−1.29245	1.66E‐22	*Smpx*	1.098375	2.32E‐08
*Esrrb*	−1.27153	1.93E‐18	*Lsamp*	1.098119	2.34E‐17
*Serpinb5*	−1.26163	3.17E‐08	*Fermt1*	1.097341	5.40E‐19
*Ptk2b*	−1.25885	2.54E‐13	*Anxa5*	1.087628	4.01E‐24
*Pcdh19*	−1.24398	1.66E‐09	*Lhfp*	1.087242	5.07E‐16
*Rgcc*	−1.18559	4.31E‐25	*Mecom*	1.082385	9.35E‐11
*Pcdh11x*	−1.17733	2.20E‐17	*Tulp2*	1.073672	7.04E‐11
*Nr1h5*	−1.16182	3.13E‐08	*Aldh1a1*	1.0728	3.31E‐17
*Tac1*	−1.14629	3.57E‐18	*Clrn1*	1.069625	2.14E‐14
*Tmprss6*	−1.14532	6.83E‐12	*Prss33*	1.068649	6.17E‐13
*Itgb6*	−1.13618	1.23E‐12	*Prdm1*	1.052381	9.71E‐09
*Nr2e1*	−1.11374	3.46E‐07	*Zfp385c*	1.045068	2.07E‐14
*Lrrtm3*	−1.11234	2.41E‐07	*Jade2*	1.033174	3.02E‐07
*Vstm2a*	−1.10418	9.18E‐07	*Cthrc1*	1.016233	2.51E‐13
*Prr15l*	−1.10112	1.03E‐14	*Fam196a*	0.995588	8.63E‐15
*Spock1*	−1.09519	4.39E‐35	*Tmem59l*	0.986324	1.21E‐07
*Cnga2*	−1.09179	1.42E‐12	*S100a1*	0.980682	1.00E‐05
*Ehf*	−1.07324	4.44E‐06	*Prss23*	0.97987	4.43E‐11
*B3galt2*	−1.02835	1.32E‐06	*Nnat*	0.972767	8.53E‐21
*Frzb*	−1.01417	1.60E‐23	*Tlr2*	0.965138	1.31E‐14
*Ltbp4*	−1.00191	4.25E‐12	*Myo7a*	0.93987	1.00E‐09
*Cyp26c1*	−0.99843	2.76E‐14	*Rims2*	0.928665	6.96E‐10
*Pcolce2*	−0.99366	5.07E‐05	*Thsd7b*	0.928559	2.17E‐05
*Vwc2*	−0.9856	8.68E‐11	*Dlx2*	0.919608	2.72E‐12
*D930020B18Rik*	−0.97348	1.01E‐06	*Mafb*	0.919096	2.53E‐07
*Gpr37*	−0.95309	1.77E‐05	*Cdh6*	0.918861	9.76E‐08
*Zdhhc14*	−0.95026	4.12E‐16	*Sgk1*	0.91884	0.000133
*Ntng1*	−0.93912	1.72E‐08	*Dapl1*	0.897578	2.01E‐05
*Frk*	−0.93653	7.63E‐17	*Cbln1*	0.889931	1.67E‐07
*Dennd4a*	−0.93638	8.38E‐12	*Rora*	0.889867	5.98E‐10

**FIGURE 7:**
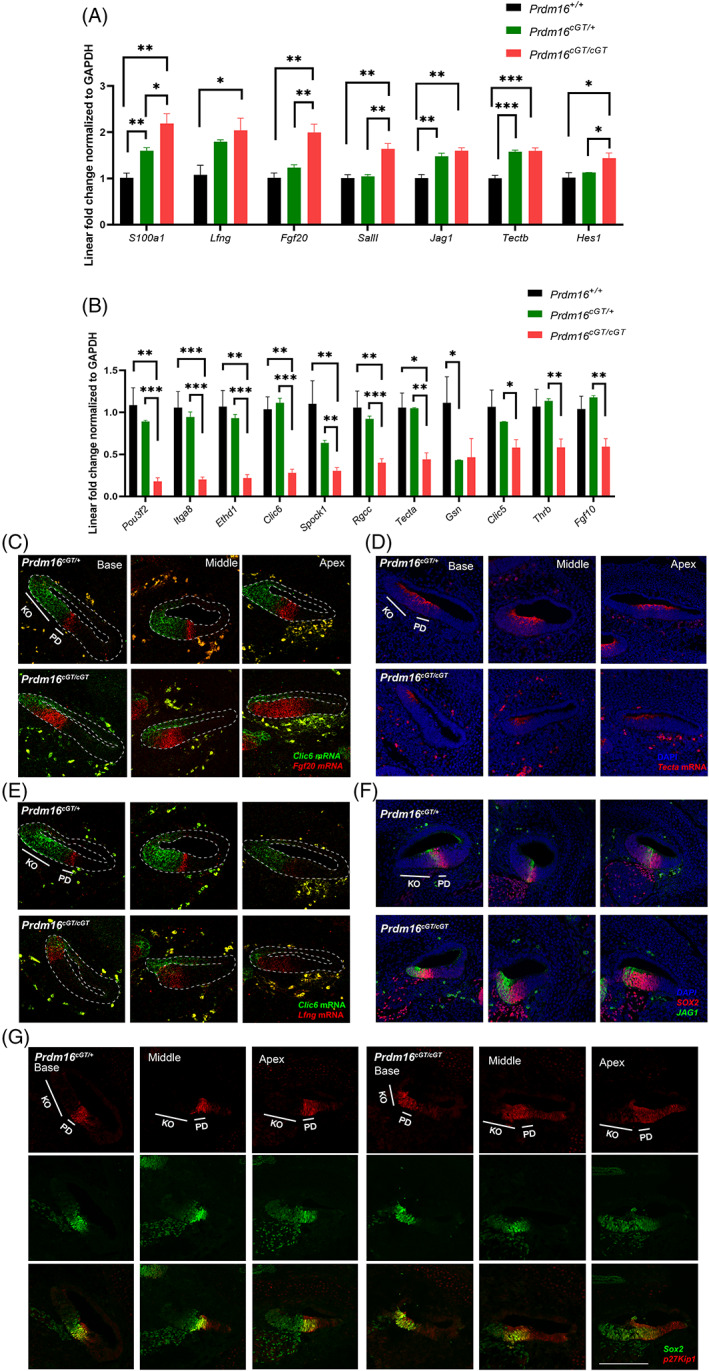
Validation of DEGs in *Prdm16* ^*cGT*^ null cochleae vs controls. Real‐time qRT‐PCR showing linear fold change in gene expression of select upregulated genes (A) and downregulated genes (B) normalized to GAPDH expression (*n* = 4 per group, Mean ± SD, multiple Student's *t*tests, *P* value * < 0.05, ** < 0.01, *** < 0.001). (C–E) RNA‐FISH of E14.5 cochlear sections showing DAPI, *Clic6* mRNA (KO marker), *Fgf20* mRNA (prosensory domain marker), *Tecta* mRNA (marker of both KO and PD) and *Lfng* mRNA (marker of the boundary between PD and KO) showing expanded domain of prosensory gene expression and downregulation of KO‐specific genes in the KO of *Prdm16* ^*cGT*^ null cochlea. (F) Immunostaining of E14.5 cochlear sections for JAG1 (green), SOX2 (red) and DAPI showing expanded JAG1 expression within the KO in *Prdm16* ^*cGT*^ null cochleae. (G) Immunostaining of E14.5 cochlear sections for p27Kip1 (red), SOX2 (green) showing expanded p27Kip1 expression within the KO in *Prdm16* ^*cGT*^ null cochleae. KO, Kölliker's organ; PD, prosensory domain (scale bar = 100 μm).

To validate the differentially expressed genes in *Prdm16* ^*cGT*^ null cochlea, quantitative real time PCR (qPCR) was performed using quadruplicate biological replicates per genotype (null, heterozygous and WT) to validate a subset of differentially expressed genes identified from RNA sequencing. We validated the upregulation of *S100a1, Lfng, Fgf20, Sall1, Jag1, Tectb*, and *Hes1* and downregulation of *Pou3f2, Itga8, Ethd1, Clic6, Sporck1, Rgcc, tecta, Gsn, Clic5, Thrb*, and *Fgf10* (N = 4, Student's *t*‐test *P* value * < 0.05, ** < 0.01, and *** < 0.001) (Figure 7A,B). We extended the validation of these genes by analyzing their domains of expression using mRNA‐FISH and immunostaining on three independent samples per genotype group. *Jag1*, *Fgf20*, *p27Kip1*, and *Lfng* showed expanded expression domains beyond the prosensory region to include parts of the KO (Figure 7C‐G). We also observed downregulation of *Clic6* and *Tecta* expression within the KO in *Prdm16* ^*cGT*^ null cochlea (Figure 7C,D). Taken together, *Prdm16* loss results in the downregulation of KO‐specific genes and upregulation of prosensory markers within the KO domain.

## 3 DISCUSSION

This work identifies *Prdm16* as a novel marker of KO during mouse cochlear development. Its expression is consistent within KO epithelial cells throughout cochlear development and early postnatal development; therefore, it can be utilized as a reliable marker of this cell population that improves upon currently used KO markers, *Fgf10* or *Tecta* ^32^ that are also expressed within the developing prosensory domain during early cochlear development, whereas *Prdm16* expression domain stops at the border of the prosensory domain. Additionally, we identified *Prdm16* expression within the stria vascularis and interdental cells during cochlear development. *Prdm16* ^*cGT*^ null cochleae are shorter, exhibit decreased proliferation of KO cells and increased density of both HCs and SCs within the apical turn. This demonstrates that *Prdm16* is necessary for the proliferation of KO epithelial cells. Proliferation of these cells is subsequently required for normal lengthening of the apical turn and to achieve normal HC and SC density within the organ of Corti. Cochlear lengthening during development is dependent upon multiple mechanisms, including convergent extension,^36^ radial intercalation and cellular growth.^37^ Due to KO location on the modiolar side of the developing cochlear duct, we demonstrate that KO lengthening is permissive to cochlear lengthening during development.

The absence of spiral limbus and the subsequent loss of modiolar anchorage of the TM in P0 *Prdm16* ^*cGT*^ null cochlea provide new evidence for the involvement of *Prdm16* in spiral limbus development and thereby correct TM anchorage. Additionally, we observed fragmentation of TM in *Prdm16* ^*cGT*^ null cochlea. Given previous evidence of KO involvement in TM formation,^3^, ^38^, ^39^ and the hypoplastic KO phenotype observed in *Prdm16* ^*cGT*^ null cochlea, we suggest that *Prdm16* loss impacts TM formation. Most of the histological preparations described in our study used dehydrated/rehydrated specimens, which makes it hard to analyze the morphology of TM. Future studies utilizing fresh cochlear preparations and subsequent μCT scanning are needed to better study TM morphology in *Prdm16* ^*cGT*^ null cochlea. Proper development of TM, assembly of its components, and its maintenance are essential for hearing, as evident by loss of function gene mutations in different genes that code for TM proteins (eg, α‐tectorin) and result in hearing impairment in mice and human.^40^, ^41^, ^42^ Given the loss of modiolar anchorage of TM and its fragmentation at birth, we anticipate hearing deficits in *Prdm16* ^*cGT*^ null mutants if they were able to survive postnatally. To this end, we are currently generating cochlear‐specific *Prdm16* conditional mutants to test this hypothesis.

The presence of ectopic HCs within the KO in *Prdm16* ^*cGT*^ null cochlea is indicative of a role of *Prdm16* in repressing HC fate in KO. The morphology of ectopic HCs is consistent with type I vestibular HCs, which are characterized by pear‐shaped cell bodies innervated by a calyx afferent surrounding the basolateral membrane^43^ and cylindrically arranged stereocilia bundles.^44^ These ectopic HCs were scarce and showed variability across samples, yet all ectopic HCs were innervated from the spiral ganglion. Our findings are in line with multiple previous studies that showed KO epithelial cell capacity to generate sensory HCs.^9^, ^10^, ^11^, ^45^ Since few KO cells turned into ectopic HCs in *Prdm16* ^*cGT*^ null cochlea, we project that *Prdm16* is not the sole regulator of KO sensory competence. Other members of the PRDM family, including *Prdm1* and *Mecom*, showed compensatory upregulation in *Prdm16* ^*cGT*^ null cochleae, and this may explain why only a few KO cells changed fates to form ectopic HCs.

To further understand the molecular mechanisms underlying the role for *Prdm16* during cochlea development, we performed gene expression analysis in *Prdm16* ^*cGT*^ null cochlea and identified that multiple sensory domain specific genes are upregulated in KO cells, including *Fgf20*, *Lfng*, *p27Kip1*, and *Jag1*.^29^, ^30^, ^31^ In‐situ and immuno‐expression analyses showed expanded domains of expression of *Fgf20*, *Lfng, p27Kip1*, and *Jag1* to include parts of the KO at E14.5 in *Prdm16* ^*cGT*^ null cochlea vs controls. *Jag1* and *Lfng* have been shown to be initially expressed within KO^46^ before becoming restricted to the differentiating prosensory domain around E14.5.^47^ In *Prdm16* ^*cGT*^ null cochlea, such restriction was not evident, indicating a role for *Prdm16* in regulating their expression. Additionally, KO‐specific genes are downregulated in *Prdm16* ^*cGT*^ null cochlea, including *Clic5*, *Clic6*, *Gsn* and *Fgf10*. Taken together, *Prdm16* is required to inhibit prosensory‐specific genes and maintain the KO‐specific genes during KO development. Our observation of expanded prosensory domain at the expense of KO along with ectopic expression of prosensory genes, including several Notch signaling pathway genes, in the KO demonstrates a role of *Prdm16* in repressing these genes in KO epithelial cells to define the boundary between the KO and the prosensory domain of the cochlea. Since Notch signaling is responsible for specifying the prosensory domain through lateral induction during cochlear development,^48^ upregulation of Notch pathway genes secondary to *Prdm16* loss, and subsequently the production of ectopic HCs in the KO, points to a role for *Prdm16* in repressing Notch signaling within the KO. Previous studies found an interplay between *Prdm16* and Notch signaling in different systems. *Prdm16* modulates Notch signaling during arterial specification.^49^, ^50^ Loss of the Notch target genes, *Hes1, 3 & 5* cause downregulation of *Prdm16* expression in the ventricular zone of the developing telencephalon.^51^ *Prdm16* has also been demonstrated to be a key regulator in the cell fate choice between skeletal muscle and brown adipose in common progenitors during embryogenesis and also influences beige adipose function and its metabolic function postnatally.^20^, ^52^, ^53^ The established role for *Prdm16* and Notch signaling in these systems and our data pertaining to sensory specification of the inner ear suggest that *Prdm16* is involved in the modulation of Notch signaling in a variety of cellular contexts.

Additionally, gene expression analysis shows downregulation of *Tecta* and upregulation of *Tectb* in *Prdm16* ^*cGT*^ null cochlea vs control. α‐tectorin (TECTA) and β‐tectorin (TECTB) are essential proteins in the structure of the TM,^54^ and mouse mutants for α‐tectorin exhibit abnormal TM and hearing loss.^40^, ^55^ Our scRNA sequencing data as well as mRNA FISH data shows that *Tecta* mRNA is expressed by KO cells (Figures 1A and 7C), and the TM phenotype in *Prdm16* ^*cGT*^ null cochlea phenocopies that observed in α‐tectorin mutant cochlea.^55^ Taken together, we predict that the loss of KO in *Prdm16* ^*cGT*^ null cochlea results in dysregulation of tectorin subunit expression that results in abnormal TM morphology. Postnatal studies in *Prdm16* conditional knockouts to evaluate TM defects will provide insight into the impact of tectorin dysregulation.

Few studies have examined the development of KO and its derivatives in mammalian cochlea,^4^, ^5^, ^6^, ^7^, ^56^ and we believe that utilizing *Prdm16* as a novel KO marker and exploring its role as a regulator of this cell population will provide new insight into the development and function of KO in mammalian cochlea. Our work sets the stage for follow up studies that will further investigate a potential role for *Prdm16* in human hearing, including the possibility of providing insight into its contribution to the underlying etiology of hearing impairment that is a common component of the 1p36 deletion syndrome phenotype.

## 4 EXPERIMENTAL PROCEDURES

### 4.1 Animals

This study was carried out in accordance with the recommendations in the Guide for the Care and Use of Laboratory Animals of the National Institutes of Health. The protocol was approved by the Midwestern University Institutional Animal Care and Use Committee. All efforts were made to minimize animal suffering. For PRDM16 expression analysis, wildtype (WT) C57BL/6J (Jax: 000664) mice were timed mated. Breeding females were checked daily for presence of post‐copulatory vaginal plugs, and if present, the developmental stage of the litter was considered embryonic day (E) 0.5. At the time points of interest, pregnant female mice were euthanized, and embryos were collected. The *Prdm16* ^*cGT*^ mutant mouse strain was previously reported.^33^ *Prdm16* ^*cGT/+*^ male and female mice were timed mated to generate *Prdm16* ^*cGT*^ null embryos (*Prdm16* ^*cGT/cGT*^), heterozygote (*Prdm16* ^*cGT/+*^), and WT (*Prdm16* ^+/+^) littermate controls. The *Prdm16* ^*cGT/+*^ strain was maintained on an FVB/NJ inbred strain background (Jax: 001800).

### 4.2 Single cell RNA sequencing (ScRNA seq)

One WT pregnant female was euthanized at E14.5, and cochleae were dissected from three pups of either sex in sterile cold HBSS, then placed in dispase (1 U/mL) in DMEM/F‐12 (Stem cell Technologies) for 15 min at 37°C. Following the incubation period, the lateral wall, and mesenchymal cells were dissected away to isolate the cochlear duct. Cochlear ducts from three pups were combined in a single tube and incubated in 0.25% trypsin‐EDTA for 15 min at 37°C with gentle trituration every 5 min, then trypsin was inactivated by adding an equal volume of DMEM/F‐12. Dissociated cells were then passed through a 30 μm strainer to exclude cell aggregates, pelleted at 300×*g* and then resuspended in 100 μl of cold PBS supplemented with 1% fetal bovine serum (FBS, Gibco: 26140079). Single cells were captured and lysed, and mRNAs were reverse transcribed into cDNAs using a 10X Genomics Chromium Controller at University of Nebraska Medical Center Sequencing core. cDNA libraries were prepared using Chromium Single Cell 3′ Reagents according to the manufacturer's instructions. Libraries were sequenced on an Illumina NextSeq to generate 60 bp of sequence to identify transcript identity. Sequences were aligned to the Ensembl mouse MM10 assembly using Cell Ranger 2.1.1 analysis software (10× Genomics). Processing of the Cell Ranger output data was done with Loupe Browser v.5 (10× Genomics). Gene expression‐based clustering information for the cells, including t‐SNE and UMAP projections and differential gene expression was done utilizing Loupe Browser v.5. The raw data from the ScRNA sequencing of E14.5 cochlear duct is deposited in gEAR database^57^ and can be accessed through the following link: https://umgear.org/p?s=ba412295&g=sox2

### 4.3 Immunostaining

WT C57BL/6J embryos, *Prdm16* ^*cGT*^ null embryos (*Prdm16* ^*cGT/cGT*^), and littermate heterozygote controls (*Prdm16* ^*cGT/+*^) at different developmental time points were collected in cold PBS, then dissected to remove skin, cranial vault, brain tissue and mandible. The skull base was then fixed with 4% paraformaldehyde overnight at 4°C then washed with PBS three times. For cryosection immunostaining, samples were washed in 10%, 20%, and 30% sucrose solutions at 4°C then embedded in OCT, and frozen on dry ice then stored at −80°C until further processing. OCT‐embedded samples were serially sectioned horizontally (10 μm thick) at −20°C using a cryostat (Leica CM1950). The sections were mounted on positively charged microscope slides (Globe Scientific Inc) and left to dry at room temperature overnight. For paraffin section immunostaining, fixed skull bases were paraffin processed using a Sakura Tissue‐Tek VIP 5 Tissue Processor overnight, then embedded in paraffin using the Sakura Tissue‐Tek TEC Paraffin Embedding Station and 7 μm sections were collected onto slides using a Thermo Scientific Microm HM325 Rotary Microtome. Slides were then placed on 40°C plate to dry out, then washed three times in Xylene, descending grades (100%, 95%, and 70%) of ethanol for 5 min each. Antigen retrieval was performed by boiling slides in Citrate Buffer pH = 6 for 8 min at 95 to 100°C. Paraffin or cryosections were then washed in PBS, permeabilized with PBS containing 0.1% Tween 20, then blocked with PBS containing 0.1% Tween 20 and 5% normal donkey serum (Southern Biotech 0030‐01). Samples were incubated in primary antibody overnight at 4°C. Samples were then washed with PBS and incubated with a secondary antibody for 2 hours at room temperature then washed, placed on a glass microscope slide in DAPI mounting media, cover‐slipped, and photographed using a Nikon A1R confocal microscope. For whole mount immunostaining, P0 pup heads were collected, inner ears were dissected in cold PBS, fixed with 4% paraformaldehyde overnight at 4°C then washed with PBS three times. Cochlear basilar membrane was micro‐dissected, permeabilized with PBS containing 0.5% Triton, blocked with PBS containing 0.5% Triton and 5% normal donkey serum. Samples were incubated in primary antibody overnight at 4°C. Samples were then washed with PBS and incubated with a secondary antibody for 2 hours at room temperature then washed, placed on a glass microscope slide in DAPI mounting media, cover‐slipped, and photographed using a Nikon A1R confocal microscope. Primary antibodies/stains used: Phalloidin (R&D Systems, 1:100), MYO6 (Proteus, 1:200), SOX2 (R&D Systems, 1:200), JAG1 (DHSB, 1:50), PRDM16,^33^ and p27Kip1 (Invitrogen 1:200).

### 4.4 EdU proliferation assay

Pregnant females were injected with EdU (400 μg/g body weight) 2 hours before collecting the embryos. Cryosections were obtained as stated previously. Staining for incorporated EdU along with SOX2 antibody and DAPI was performed according to Click‐iT EdU Cell Proliferation Kit (Invitrogen C10340) manufacturer protocol. The stained cryosection slides were then imaged on a Nikon A1R confocal microscope.

### 4.5 Bulk RNA extraction and sequencing

RNAqueous RNA isolation kit (Invitrogen, AM1931) was used for bulk RNA extraction as per the manufacturer protocol. Briefly, E14.5 cochlear ducts from both inner ears of one embryo were microdissected in DEPC‐treated cold PBS, collected in RNA extraction buffer and homogenized (Bel‐Art 650 000 000). RNA extraction and subsequent removal of genomic DNA were performed according to the manufacturer's protocol. Three littermates per genotype were used. The yield and integrity of total RNA from microdissected samples were measured using a 2100 Bioanalyzer (Agilent Technologies). Next generation sequencing and bioinformatics analysis were performed at Northwestern Sequencing core. Briefly, TruSeq mRNA‐Seq Library Prep was used to create cDNA libraries according to manufacturer's protocol. Each library was sequenced to generate 50 base pair single reads on the Illumina HiSeq Sequencing (Illumina). The sequence reads were aligned to the mouse reference genome sequence (USCS mm10) using STAR aligner.^58^ Alignments were assembled and annotated using Cufflinks.^59^ DESeq2^60^ was used to detect differentially expressed gene transcripts. The raw data from bulk RNA sequencing of E14.5 cochlear duct is deposited in Gene expression Omnibus (GSE193046) and can be accessed through the following link: https://www.ncbi.nlm.nih.gov/geo/query/acc.cgi?acc=GSE193046

### 4.6 Pathway analysis

The database DAVID (the Database of Annotation, Visualization and Integrated Discovery)^34^, ^35^ was used for pathway analysis. The differentially expressed gene transcripts (*q* < 0.05) identified from the RNA‐Seq data were input into DAVID, which identified enriched biological pathways.

### 4.7 RNA fluorescence in situ hybridization (FISH)

Cryosections (10 μm) from E14.5 embryos were used for RNA FISH analysis following the manufacturer's protocol (Molecular instruments, HCR RNA‐FISH protocol for fresh frozen or fixed frozen tissue sections https://files.molecularinstruments.com/MI‐Protocol‐RNAFISH‐FrozenTissue‐Rev2.pdf).^61^ Probes used are included in Table S3).

### 4.8 Quantitative real time PCR

Reverse transcription of total RNA was performed using GoScript Reverse Transcription System (Promega A5001), and quantitative real time PCR (qPCR) was performed using PowerUp SYBR Green (Applied Biosytems, A25742), each according to manufacturer's protocols. Data analysis was performed using the comparative CT method,^62^ and data were normalized to detection of GAPDH RNA. Primers used are included in Table S4.

### 4.9 Data analysis and statistics

Data analysis was done using Image J software Version 1.53n.^63^ Cochlear length was measured from the tip of the apex to the base using MYO6 immunostaining as a marker for the hair cells. To measure HC and SC density, at least 400 μm regions of the base, middle, and apex of whole‐mount immunostained cochleae were counted and normalized to 100 μm. KO and stria vascularis thickness was measured using H&E‐stained sections. The thickness was measured at three different points at 25%, 50%, and 75% of the length of the structure in five consecutive 7 μm thick sections then averaged per each biological sample. For EdU proliferation assay, the percentage of KO cells incorporating EdU marker was calculated by counting all KO cells on the neural side of SOX2‐positive cells using DAPI staining, then counting the number of EdU‐positive cells within this population and calculating the percentage using five consecutive 10 μm thick sections then averaged per each biological sample. For each experiment, the numbers of samples (n) is indicated. The *P* value for difference between samples was calculated using either multiple‐testing adjusted *P* value (for differential expression using DESeq2^60^) or a multiple unpaired two‐tailed Student's *t*‐test (for length, thickness, cell density, percentage of proliferation quantification, and qRT‐PCR), and *P* < 0.05 was considered as significant.

## AUTHOR CONTRIBUTIONS

**Michael Ebeid:** Conceptualization (lead); data curation (lead); formal analysis (lead); funding acquisition (lead); investigation (lead); methodology (equal); project administration (lead); resources (lead); software (lead); supervision (lead); validation (lead); visualization (equal); writing – original draft (lead); writing – review and editing (lead). **Kathy Barnas:** Conceptualization (equal); data curation (equal); formal analysis (equal); investigation (equal); methodology (equal); validation (equal); visualization (equal); writing – review and editing (equal). **Hongji Zhang:** Investigation (equal); methodology (equal); project administration (equal); validation (equal); writing – review and editing (equal). **Amal Yaghmour:** Formal analysis (equal); investigation (equal); methodology (equal); writing – review and editing (equal). **Gabriele Noreikaite:** Formal analysis (equal); investigation (equal); methodology (equal); writing – review and editing (equal). **Bryan C. Bjork:** Conceptualization (equal); methodology (equal); resources (equal); supervision (equal); writing – review and editing (equal).

## Supporting information


**TABLE S1** Complete list of differentially expressed genes within each population of cochlear duct cells at E14.5 from Wilde‐type mouse using single cell RNA sequencing.

**TABLE S2** Complete list of differentially expressed genes in Prdm16 cGT null cochlear duct cells at E14.5 compared to littermate WT controls using bulk RNA sequencing.

**TABLE S3** List of RNA FISH probes name, size, accession numbers and linked fluorophores.

**TABLE S4** List of quantitative real time PCR primer sequences.

## References

[1] Morsli H , Choo D , Ryan A , Johnson R , Wu DK . Development of the mouse inner ear and origin of its sensory organs. J Neurosci. 1998;18(9):3327‐3335. doi:10.1523/jneurosci.18-09-03327.1998 9547240 PMC6792659

[2] Kelley MW , Xu XM , Wagner MA , Warchol ME , Corwin JT . The developing organ of Corti contains retinoic acid and forms supernumerary hair cells in response to exogenous retinoic acid in culture. Development Dec 1993;119(4):1041–53. 8306874 10.1242/dev.119.4.1041

[3] Hinojosa R. A note on development of Corti's organ. Acta Otolaryngol Sep‐Oct 1977;84(3–4):238–51. doi:10.3109/00016487709123963 906817

[4] Uziel A , Gabrion J , Ohresser M , Legrand C . Effects of hypothyroidism on the structural development of the organ of Corti in the rat. Acta Otolaryngol Nov‐Dec 1981;92(5–6):469–80. doi:10.3109/00016488109133286 7315266

[5] Legrand C , Bréhier A , Clavel MC , Thomasset M , Rabié A . Cholecalcin (28‐kDa CaBP) in the rat cochlea. Development in normal and hypothyroid animals. An immunocytochemical study. Brain Res 1988;466(1):121–9. doi:10.1016/0165-3806(88)90090-9 3342324

[6] Anniko M . Embryogenesis of the mammalian inner ear. III. Formation of the tectorial membrane of the CBA/CBA mouse in vivo and in vitro. Anat Embryol (Berl). 1980;160(3):301‐313. doi:10.1007/bf00305110 7457923

[7] Tritsch NX , Yi E , Gale JE , Glowatzki E , Bergles DE . The origin of spontaneous activity in the developing auditory system. Nature. 2007;450(7166):50‐55. doi:10.1038/nature06233 17972875

[8] Babola TA , Li S , Wang Z , Kersbergen CJ , Elgoyhen AB , Coate TM , Bergles DE Purinergic signaling controls spontaneous activity in the auditory system throughout early development. J Neurosci Jan 27 2021;41(4):594–612. doi:10.1523/jneurosci.2178-20.2020 33303678 PMC7842760

[9] Zheng JL , Gao WQ . Overexpression of Math1 induces robust production of extra hair cells in postnatal rat inner ears. Nat Neurosci Jun 2000;3(6):580–6. doi:10.1038/75753 10816314

[10] Kelly MC , Chang Q , Pan A , Lin X , Chen P . Atoh1 directs the formation of sensory mosaics and induces cell proliferation in the postnatal mammalian cochlea in vivo. J Neurosci. 2012;32(19):6699‐6710. doi:10.1523/jneurosci.5420-11.2012 22573692 PMC3477623

[11] Puligilla C , Kelley MW . Dual role for Sox2 in specification of sensory competence and regulation of Atoh1 function. Dev Neurobiol Jan 2017;77(1):3–13. doi:10.1002/dneu.22401 27203669 PMC5116417

[12] Basch ML , Brown RM 2nd , Jen H‐I , et al. Fine‐tuning of notch signaling sets the boundary of the organ of Corti and establishes sensory cell fates. Elife. 2016;5:e19921. doi:10.7554/eLife.19921 27966429 PMC5215100

[13] Driver EC , Pryor SP , Hill P , et al. Hedgehog signaling regulates sensory cell formation and auditory function in mice and humans. J Neurosci. 2008;28(29):7350‐7358. doi:10.1523/JNEUROSCI.0312-08.2008 18632939 PMC2581462

[14] Fumasoni I , Meani N , Rambaldi D , Scafetta G , Alcalay M , Ciccarelli FD . Family expansion and gene rearrangements contributed to the functional specialization of PRDM genes in vertebrates. BMC Evol Biol. 2007;7(1):187. doi:10.1186/1471-2148-7-187 17916234 PMC2082429

[15] Ishibashi J , Seale P . Functions of Prdm16 in thermogenic fat cells. Temperature (Austin). 2015;2(1):65‐72. doi:10.4161/23328940.2014.974444 27227007 PMC4843880

[16] Chuikov S , Levi BP , Smith ML , Morrison SJ . Prdm16 promotes stem cell maintenance in multiple tissues, partly by regulating oxidative stress. Nat Cell Biol Oct 2010;12(10):999–1006. doi:10.1038/ncb2101 20835244 PMC2948585

[17] Aguilo F , Avagyan S , Labar A , Sevilla A , Lee DF , Kumar P , Lemischka IR , Zhou BY , Snoeck HW Prdm16 is a physiologic regulator of hematopoietic stem cells. Blood May 12, 2011;117(19):5057–66. doi:10.1182/blood-2010-08-300145 21343612 PMC3109532

[18] Bjork BC , Turbe‐Doan A , Prysak M , Herron BJ , Beier DR . Prdm16 is required for normal palatogenesis in mice. Hum Mol Genet. 2010;19(5):774‐789. doi:10.1093/hmg/ddp543 20007998 PMC2816611

[19] Shull LC , Sen R , Menzel J , Goyama S , Kurokawa M , Artinger KB . The conserved and divergent roles of Prdm3 and Prdm16 in zebrafish and mouse craniofacial development. Dev Biol. 2020;461(2):132‐144. doi:10.1016/j.ydbio.2020.02.006 32044379 PMC7198358

[20] Seale P , Kajimura S , Yang W , et al. Transcriptional control of brown fat determination by PRDM16. Cell Metab. 2007;6(1):38‐54. doi:10.1016/j.cmet.2007.06.001 17618855 PMC2564846

[21] Warner DR , Wells JP , Greene RM , Pisano MM . Gene expression changes in the secondary palate and mandible of Prdm16(−/−) mice. Cell Tissue Res. 2013;351(3):445‐452. doi:10.1007/s00441-012-1525-2 23149718 PMC3584240

[22] Harms MJ , Lim HW , Ho Y , et al. PRDM16 binds MED1 and controls chromatin architecture to determine a brown fat transcriptional program. Genes Dev. 2015;29(3):298‐307. doi:10.1101/gad.252734.114 25644604 PMC4318146

[23] Shaffer LG , Heilstedt HA . Terminal deletion of 1p36. The Lancet. 2001;358:S9. doi:10.1016/S0140-6736(01)07022-2 11784558

[24] Heilstedt HA , Ballif BC , Howard LA , Kashork CD , Shaffer LG . Population data suggest that deletions of 1p36 are a relatively common chromosome abnormality. Clin Genet. 2003;64(4):310‐316. doi:10.1034/j.1399-0004.2003.00126.x 12974736

[25] Trowe MO , Maier H , Schweizer M , Kispert A . Deafness in mice lacking the T‐box transcription factor Tbx18 in otic fibrocytes. Development. 2008;135(9):1725‐1734. doi:10.1242/dev.014043 18353863

[26] Phippard D , Heydemann A , Lechner M , et al. Changes in the subcellular localization of the Brn4 gene product precede mesenchymal remodeling of the otic capsule. Hear Res. 1998;120(1–2):77‐85. doi:10.1016/s0378-5955(98)00059-8 9667433

[27] Pirvola U , Zhang X , Mantela J , Ornitz DM , Ylikoski J . Fgf9 signaling regulates inner ear morphogenesis through epithelial–mesenchymal interactions. Dev Biol. 2004;273(2):350‐360. doi:10.1016/j.ydbio.2004.06.010 15328018

[28] Morsli H , Tuorto F , Choo D , Postiglione MP , Simeone A , Wu DK . Otx1 and Otx2 activities are required for the normal development of the mouse inner ear. Development. 1999;126(11):2335‐2343. 10225993 10.1242/dev.126.11.2335

[29] Morrison A , Hodgetts C , Gossler A , Hrabé de Angelis M , Lewis J . Expression of Delta1 and Serrate1 (Jagged1) in the mouse inner ear. Mech Dev. 1999;84(1–2):169‐172. doi:10.1016/s0925-4773(99)00066-0 10473135

[30] Hume CR , Bratt DL , Oesterle EC . Expression of LHX3 and SOX2 during mouse inner ear development. Gene Expr Patterns. 2007;7(7):798‐807. doi:10.1016/j.modgep.2007.05.002 17604700 PMC2043117

[31] Hayashi T , Kokubo H , Hartman BH , Ray CA , Reh TA , Bermingham‐McDonogh O . Hesr1 and Hesr2 may act as early effectors of notch signaling in the developing cochlea. Dev Biol. 2008;316(1):87‐99. doi:10.1016/j.ydbio.2008.01.006 18291358 PMC2362132

[32] Pauley S , Wright TJ , Pirvola U , Ornitz D , Beisel K , Fritzsch B . Expression and function of FGF10 in mammalian inner ear development. Dev Dyn Jun 2003;227(2):203–15. doi:10.1002/dvdy.10297 12761848 PMC3904739

[33] Strassman A , Schnütgen F , Dai Q , et al. Generation of a multipurpose Prdm16 mouse allele by targeted gene trapping. Dis Model Mech. 2017;10(7):909‐922. doi:10.1242/dmm.029561 28424158 PMC5536910

[34] Huang DW , Sherman BT , Lempicki RA . Systematic and integrative analysis of large gene lists using DAVID bioinformatics resources. Nat Protoc. 2009;4(1):44‐57. doi:10.1038/nprot.2008.211 19131956

[35] Huang da W , Sherman BT , Lempicki RA . Bioinformatics enrichment tools: paths toward the comprehensive functional analysis of large gene lists. Nucleic Acids Res. 2009;37(1):1‐13. doi:10.1093/nar/gkn923 19033363 PMC2615629

[36] Chen P , Johnson JE , Zoghbi HY , Segil N . The role of Math1 in inner ear development: uncoupling the establishment of the sensory primordium from hair cell fate determination. Development. 2002;129(10):2495‐2505. 11973280 10.1242/dev.129.10.2495

[37] Driver EC , Northrop A , Kelley MW . Cell migration, intercalation and growth regulate mammalian cochlear extension. Development. 2017;144(20):3766‐3776. doi:10.1242/dev.151761 28870992 PMC5675446

[38] Lim DJ , Anniko M . Developmental morphology of the mouse inner ear. A scanning electron microscopic observation. Acta Otolaryngol Suppl. 1985;422:1‐69. 3877398

[39] Zine A , Romand R . Development of the auditory receptors of the rat: a SEM study. Brain Res. 1996;721(1–2):49‐58. doi:10.1016/0006-8993(96)00147-3 8793083

[40] Legan PK , Goodyear RJ , Morín M , et al. Three deaf mice: mouse models for TECTA‐based human hereditary deafness reveal domain‐specific structural phenotypes in the tectorial membrane. Hum Mol Genet. 2014;23(10):2551‐2568. doi:10.1093/hmg/ddt646 24363064 PMC3990158

[41] Xia A , Gao SS , Yuan T , et al. Deficient forward transduction and enhanced reverse transduction in the alpha tectorin C1509G human hearing loss mutation. Dis Model Mech. 2010;3(3–4):209‐223. doi:10.1242/dmm.004135 20142329 PMC2869304

[42] Verhoeven K , Laer LV , Kirschhofer K , et al. Mutations in the human α‐tectorin gene cause autosomal dominant non‐syndromic hearing impairment. Nat Genet. 1998;9(1):60‐62. doi:10.1038/ng0598-60 9590290

[43] Meredith FL , Rennie KJ . Channeling your inner ear potassium: K(+) channels in vestibular hair cells. Hear Res. 2016;338:40‐51. doi:10.1016/j.heares.2016.01.015 26836968

[44] Assad JA , Corey DP . An active motor model for adaptation by vertebrate hair cells. J Neurosci. 1992;12(9):3291‐3309. doi:10.1523/JNEUROSCI.12-09-03291.1992 1527581 PMC6575747

[45] Ahmed M , Wong EY , Sun J , Xu J , Wang F , Xu PX . Eya1‐Six1 interaction is sufficient to induce hair cell fate in the cochlea by activating Atoh1 expression in cooperation with Sox2. Dev Cell. 2012;22(2):377‐390. doi:10.1016/j.devcel.2011.12.006 22340499 PMC3285434

[46] Ohyama T , Basch ML , Mishina Y , Lyons KM , Segil N , Groves AK . BMP signaling is necessary for patterning the sensory and nonsensory regions of the developing mammalian cochlea. J Neurosci. 2010;30(45):15044‐15051. doi:10.1523/jneurosci.3547-10.2010 21068310 PMC3074492

[47] Kiernan AE , Xu J , Gridley T . The notch ligand JAG1 is required for sensory progenitor development in the mammalian inner ear. PLoS Genetics. 2006;2(1):e4. doi:10.1371/journal.pgen.0020004 16410827 PMC1326221

[48] Hartman Byron H , Reh Thomas A , Bermingham‐McDonogh O . Notch signaling specifies prosensory domains via lateral induction in the developing mammalian inner ear. Proceedings of the National Academy of Sciences. 2010;107(36):15792‐15797. doi:10.1073/pnas.1002827107 PMC2936601 20798046

[49] Beerens M , Van Wauwe J , Craps S , et al. Prdm16 and notch functionally and physically interact during artery development. bioRxiv. 2021;471275. doi:10.1101/2021.12.05.471275

[50] Aranguren XL , Agirre X , Beerens M , et al. Unraveling a novel transcription factor code determining the human arterial‐specific endothelial cell signature. Blood. 2013;122(24):3982‐3992. doi:10.1182/blood-2013-02-483255 24108462

[51] Kinameri E , Inoue T , Aruga J , et al. Prdm proto‐oncogene transcription factor family expression and interaction with the Notch‐Hes pathway in mouse neurogenesis. PLoS One. 2008;3(12):e3859. doi:10.1371/journal.pone.0003859 19050759 PMC2585159

[52] Seale P , Bjork B , Yang W , et al. PRDM16 controls a brown fat/skeletal muscle switch. Nature. 2008;454(7207):961‐967. doi:10.1038/nature07182 18719582 PMC2583329

[53] Cohen P , Levy JD , Zhang Y , et al. Ablation of PRDM16 and beige adipose causes metabolic dysfunction and a subcutaneous to visceral fat switch. Cell. 2014;156(1–2):304‐316. doi:10.1016/j.cell.2013.12.021 24439384 PMC3922400

[54] Richardson GP , Lukashkin AN , Russell IJ . The tectorial membrane: one slice of a complex cochlear sandwich. Curr Opin Otolaryngol Head Neck Surg. 2008;16(5):458‐464. doi:10.1097/MOO.0b013e32830e20c4 18797289 PMC2874155

[55] Kim D‐K , Kim JA , Park J , Niazi A , Almishaal A , Park S . The release of surface‐anchored α‐tectorin, an apical extracellular matrix protein, mediates tectorial membrane organization. Sci Adv. 2019;5(11):eaay6300. doi:10.1126/sciadv.aay6300 31807709 PMC6881170

[56] Dayaratne MWN , Vlajkovic SM , Lipski J , Thorne PR . Kölliker's organ and the development of spontaneous activity in the auditory system: implications for hearing dysfunction. Biomed Res Int. 2014;2014:367939. doi:10.1155/2014/367939 25210710 PMC4156998

[57] Orvis J , Gottfried B , Kancherla J , et al. gEAR: gene expression analysis resource portal for community‐driven, multi‐omic data exploration. Nat Methods. 2021;18(8):843‐844. doi:10.1038/s41592-021-01200-9 34172972 PMC8996439

[58] Dobin A , Davis CA , Schlesinger F , et al. STAR: ultrafast universal RNA‐seq aligner. Bioinformatics. 2013;29(1):15‐21. doi:10.1093/bioinformatics/bts635 23104886 PMC3530905

[59] Trapnell C , Williams BA , Pertea G , et al. Transcript assembly and quantification by RNA‐Seq reveals unannotated transcripts and isoform switching during cell differentiation. Nat Biotechnol. 2010;28(5):511‐515. doi:10.1038/nbt.1621 20436464 PMC3146043

[60] Anders S , Huber W . Differential expression analysis for sequence count data. Genome Biol. 2010;11(10):R106. doi:10.1186/gb-2010-11-10-r106 20979621 PMC3218662

[61] Choi HMT , Schwarzkopf M , Fornace ME , et al. Third‐generation in situ hybridization chain reaction: multiplexed, quantitative, sensitive, versatile, robust. Development. 2018;145(12):dev165753. doi:10.1242/dev.165753 29945988 PMC6031405

[62] Schmittgen TD , Livak KJ . Analyzing real‐time PCR data by the comparative CT method. Nat Protoc. 2008;3(6):1101‐1108. doi:10.1038/nprot.2008.73 18546601

[63] Schindelin J , Arganda‐Carreras I , Frise E , et al. Fiji: an open‐source platform for biological‐image analysis. Nat Methods. 2012;9(7):676‐682. doi:10.1038/nmeth.2019 22743772 PMC3855844

